# Identifying the role of vision transformer for skin cancer—A scoping review

**DOI:** 10.3389/frai.2023.1202990

**Published:** 2023-07-17

**Authors:** Sulaiman Khan, Hazrat Ali, Zubair Shah

**Affiliations:** College of Science and Engineering, Hamad Bin Khalifa University, Qatar Foundation, Doha, Qatar

**Keywords:** scoping review, lesion segmentation, skin cancer, melanocytic lesion, vision transformers

## Abstract

**Introduction:**

Detecting and accurately diagnosing early melanocytic lesions is challenging due to extensive intra- and inter-observer variabilities. Dermoscopy images are widely used to identify and study skin cancer, but the blurred boundaries between lesions and besieging tissues can lead to incorrect identification. Artificial Intelligence (AI) models, including vision transformers, have been proposed as a solution, but variations in symptoms and underlying effects hinder their performance.

**Objective:**

This scoping review synthesizes and analyzes the literature that uses vision transformers for skin lesion detection.

**Methods:**

The review follows the PRISMA-ScR (Preferred Reporting Items for Systematic Reviews and Meta-Analyses Extension for Scoping Revise) guidelines. The review searched online repositories such as IEEE Xplore, Scopus, Google Scholar, and PubMed to retrieve relevant articles. After screening and pre-processing, 28 studies that fulfilled the inclusion criteria were included.

**Results and discussions:**

The review found that the use of vision transformers for skin cancer detection has rapidly increased from 2020 to 2022 and has shown outstanding performance for skin cancer detection using dermoscopy images. Along with highlighting intrinsic visual ambiguities, irregular skin lesion shapes, and many other unwanted challenges, the review also discusses the key problems that obfuscate the trustworthiness of vision transformers in skin cancer diagnosis. This review provides new insights for practitioners and researchers to understand the current state of knowledge in this specialized research domain and outlines the best segmentation techniques to identify accurate lesion boundaries and perform melanoma diagnosis. These findings will ultimately assist practitioners and researchers in making more authentic decisions promptly.

## 1. Introduction

Cancer is predicted to become the leading cause of death and the most significant obstacle to increasing life expectancy worldwide in the 21st century (World Health Organization, 2023). In 2015, the World Health Organization (WHO) estimated that cancer is the first or second leading cause of death before the age of 70 years in 91 out of 172 countries. In an additional 22 countries, it ranks as the third or fourth leading cause of death. The American Cancer Society reported that skin cancer is the most common type of cancer, with high mortalities and growth rates in the US and many other countries (Xie et al., 2021). Skin cancer is the second leading cause of mortalities in the United States (Siegel et al., 2019) and a major health problem in the world. Among skin cancers, melanoma is the most malignant cancer, which caused about 9.3 million deaths and 1.20 million new cases in 2023 (Siegel et al., 2023).

Dermoscopy is a commonly used technique for observing skin disorders and distinguishing between benign and malignant skin cancers (Yu et al., 2017). Automated and precise segmentation of skin lesions in dermoscopy images is a crucial step in computer-assisted skin cancer diagnosis. Segmentation masks of the skin lesion can provide information such as location, shape, size, and other quantitative data, which can significantly enhance the accuracy and efficiency of skin cancer diagnosis (Xie et al., 2020; Ding et al., 2021). In the past, several approaches based on traditional machine learning and image processing techniques have been reported for skin lesion detection and segmentation. For example, Murugan et al. (2019) suggested support vector machine, random forest, and K-nearest neighbor classifiers accompanied by watershed segmentation technique to extract the shape, asymmetry, border, color, diameter (ABCD rule), and Gray Level Co-occurrence Matrix (GLCM) based features. Alquran et al., (Alquran et al., 2017) used SVM classifier for melanoma cancer detection using ABCD and GLCM feature maps.

The emergence of advanced deep learning and machine learning-based models has minimized the efforts for feature extraction by automatically extracting astute information from dermoscopy images and performing classification tasks accordingly. Many studies have recently developed vision transformer-based deep learning methods for skin lesion detection and skin cancer diagnosis. The Vision Transformer or Vit is a deep learning architecture that uses the Transformer architecture and is specifically designed for images and computer vision tasks (Dosovitskiy et al., 2020). Since its introduction, numerous variations and improvements to the Vision Transformer have been proposed, such as hybrid models that combine CNNs with Transformers or modifications to the self-attention mechanism to better handle spatial information. Given the popularity of vision transformer, many recent studies adopted it for skin cancer imaging applications. However, a review has yet to be published to analyze the published studies and identify research gaps accordingly systematically.

While few reviews have been reported for skin lesion detection and skin cancer diagnosis (Korotkov and Garcia, 2012; Filho et al., 2015; Oliveira et al., 2016; Pathan et al., 2018; Pereira et al., 2020; Kassem et al., 2021; Nie et al., 2022), these reviews do not include vision transformer-based methods. Table 1 identifies the difference between our review and the previously published review articles. After studying the literature, it was concluded that the published survey articles cover the topic only partially and do not include recent studies (as depicted in Table 1). Our review aims to address the gap by including the most recent research efforts on vision transformer-based methods for skin cancer. Compared to the previously published reviews, our work provides a state-of-the-art review on the topic as it specifically covers studies published after 2019. Our review offers comprehensive information for researchers about the recent progress in skin lesion detection and diagnosis. Additionally, it provides detailed information about data sources that are helpful for AI researchers to develop enhanced solutions for skin cancer applications. The following are the research questions considered for this review:

- Lesion detection and feature extraction: what common vision transformer-based techniques were developed to detect skin lesions in dermoscopy images? How multiple feature extraction techniques are used to accumulate semantic-based information (local and global features) from these images?
- Benchmark models: what different types of benchmark models are used to evaluate the performance of the vision transformer-based models?
- Vision transformer role in skin cancer detection: were vision transformers effective in enhancing cancer detection performance? How have vision transformers augmented the performance of convolutional neural networks for skin cancer detection?
- Data sources: what are the commonly used datasets for skin cancer that contributed to developing vision transformer-based models?

**Table 1:** Comparative analysis of our work to published review articles.

**References**	**Year**	**Short description of previous reviews**	**Comparative contribution with our work**
Nie et al. (2022)	2022	This paper has reviewed the literatures reported for skin lesion classification using dermoscopy images based on CNN-based transformer architectures.	Our review covers vision transformer-based approaches for classification and detection tasks of skin cancer.
Oliveira et al. (2016)	2016	This review has covered only image acquisition and segmentation techniques for skin cancer. It contains no information about vision transformers or other transformer-based models.	Our review covers vision transformer-based approaches.
Pereira et al. (2020)	2020	This review has evaluated multiple segmentation techniques for accurate lesion boundaries detection in dermoscopy images.	Our work focuses on identifying the segmentation techniques, and covers vision transformer architectures proposed for skin cancer detection.
Kassem et al. (2021)	2021	This review included various deep and shallow architectures and their capabilities for skin lesion detection and. It did not cover transformer-based methods.	Our review covers the role of vision transformers for skin cancer detection.
Pathan et al. (2018)	2018	This review analyzed the literature to identify the best feature extraction techniques for dermoscopy images. It did not cover vision transformer models and their capabilities for skin cancer.	In our review, we focus on vision transformer-based architectures reported for skin cancer detection.
Filho et al. (2015)	2015	This review assessed the literature for identifying various integrated and hand-held devices proposed for quantifying and classifying PSL. The main objective of the review was to identify studies that developed methods for diagnosis of PSLs on hand-held devices.	Our review analyzes the most recent literature for identifying the role of vision transformers for skin cancer diagnosing and detection.

This scoping review will serve as a comprehensive overview of the applications of vision transformers in skin lesion detection and diagnosis. Additionally, both researchers and practitioners will be able to use the findings of the review as evidence to make informed decisions when developing AI models for skin cancer. The remainder of the paper is organized as follows: the study protocol and methodology are described in Section 2, covering the search for the relevant studies, selection of studies, data extraction, and synthesis. Section 3 of the paper outlines the research findings of this review based on the research questions. The discussion based on the findings of the scoping review is provided in Section 4. Section 5 of the paper outlines the strengths and limitations of this review. Finally, Section 6 concludes the paper.

## 2. Methods

PRISMA-ScR guidelines are followed for this scoping review (Tricco et al., 2018). Supplementary Table 1 presents the adherence to the PRISMA-ScR guidelines. The search process and study selection steps are described below.

### 2.1. Search process

In this research work, four reputable online repositories (IEEE Xplore, Scopus, PubMed, and Google Scholar) were selected for retrieving relevant research articles. The search was conducted on January 7, 2023, and January 8, 2023. The search results were limited to the first 100 entries on Google Scholar as, beyond this point, the relevance of the studies to the topic of the review decreased significantly. Moreover, we also reviewed the reference lists of the finalized articles to identify any additional relevant studies. Our search string incorporates three major terms. Supplementary Table 2 shows the search strings. Where applicable, we used different forms of each search term and refined the search string further based on the search results and database requirements.

### 2.2. Inclusion and exclusion criteria

We included research studies that reported vision transformer-based approaches for melanocytic lesion segmentation and detection using images. We included studies published in the English language in or after the year 2017. We included research studies that use vision transformers for lesion segmentation, lesion boundaries identification, lesion detection, and semantic information/features calculation from dermoscopy images. We excluded studies that used vision transformers for medical image data other than skin cancer applications. During the process of inclusion and exclusion, we considered only primary studies and conference papers, and excluded preprints, short reviews, commentaries, editorials, and abstracts. Additionally, non-English studies were excluded. No constraints were applied on the country of publication, comparators, or outcomes related to the performance of the vision transformer models.

### 2.3. Study selection

In this study, we employed the Rayyan web-based review management tool (Ouzzani et al., 2016) for the initial screening and selection of studies. Duplicates were removed, and the remaining studies were evaluated based on their titles and abstracts. The contents of the studies that met the inclusion and exclusion criteria were then assessed for eligibility by two authors (S.K. and H.A.). Any discrepancies that arose during the study selection process were discussed and resolved among the authors, and a final agreement was reached after mutual discussion.

### 2.4. Data extraction

A data extraction sheet was prepared to retrieve all relevant information from the final included articles. This information includes the first author's name, publication year, type of article (conference paper, journal article), first author's institution and location (country), data modality, availability of data (public or private, with access link), architecture of the vision transformer model, performance validation metrics, feature extraction methods, hardware requirements, training and testing parameters, number of images used for training, testing, and validation process, different parameters used for implementation, and comparison with other benchmark models. In Supplementary Table 3, we presented a description of the extracted information. The data extraction process was performed by the authors (S.K. and H.A.), and the extracted data was reviewed and verified by the third author (Z.S). Any confusion or disagreement was resolved through mutual discussion and consensus between the authors.

### 2.5. Data synthesis

In this research work a narrative mechanism is used to synthesize the data after the data extraction process. The finalized included studies were evaluated from five different perspectives: lesion boundaries detection, vision transformer effectiveness in skin cancer detection, key challenges, data modality, and data sources. For lesion boundary detection, we focused on how vision transformers were used to achieve optimum lesion segmentation and retrieve accurate semantic-based information from dermoscopy images. Furthermore, we analyzed different transformer-based models reported for skin cancer detection. Our analysis engrossed on the genre of dermoscopy and imaging data used in the included studies, as well as the data source and its accessibility. Additionally, we examined the evaluation metrics employed by each study to assess the robustness of various transformer-based models for melanocytic lesions.

## 3. Results

### 3.1. Search results

**Figure 1:**
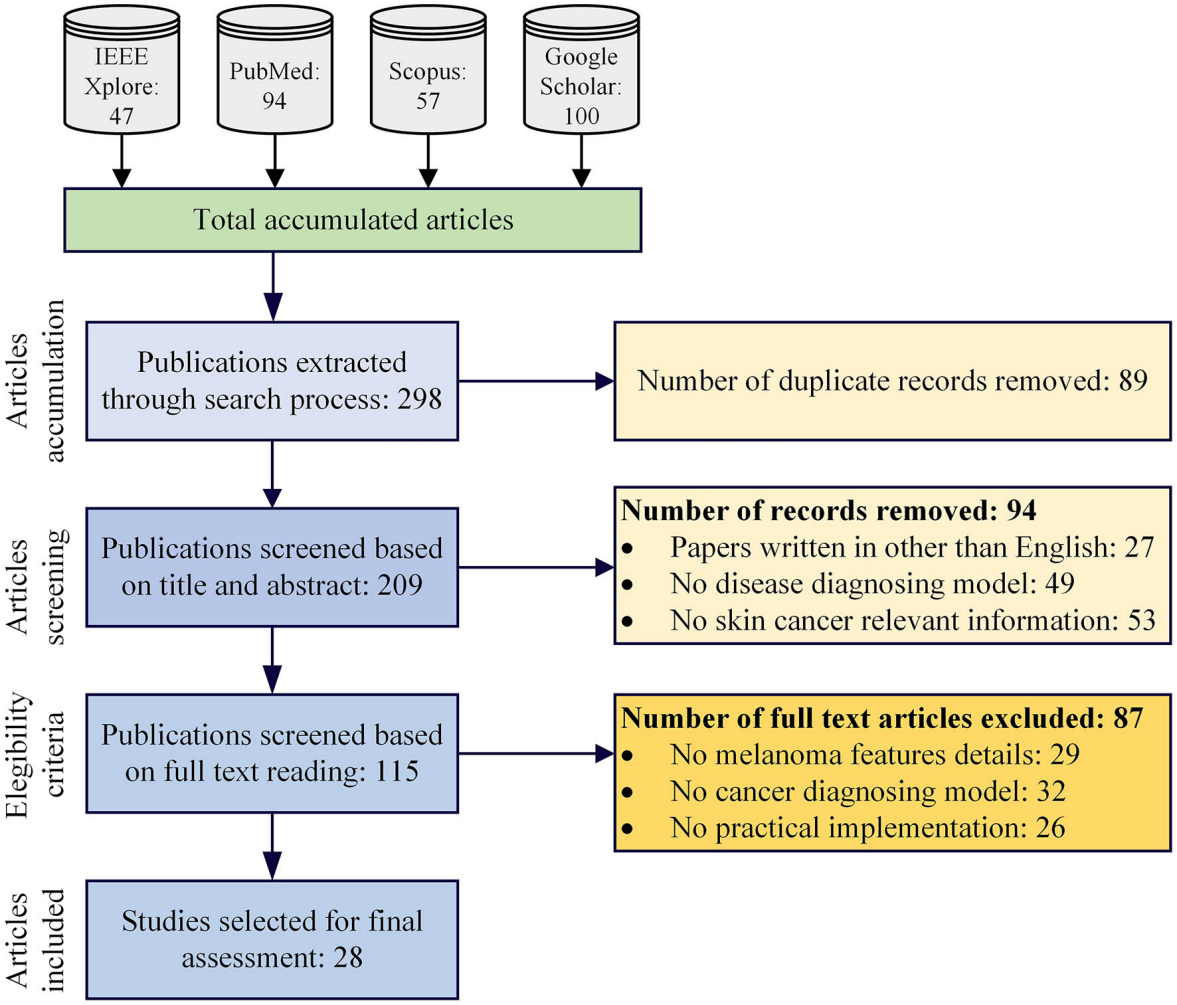
Proposed scoping review protocol.

In our initial literature search, we retrieved 298 studies related to the topic. After removing duplicate entries, we were left with 209 studies for further evaluation. Using our established inclusion and exclusion criteria (see Methods section), we screened these studies based on their abstracts and titles and selected 115 studies for full-text review. Out of these 115 studies, 87 were excluded during the full-text screening process, leaving only 28 studies that met our inclusion criteria. Laterally, these 28 articles are used for the data synthesizing and evaluation process. Figure 1 represents the overall screening and studies selection process for the proposed research work.

### 3.2. Demographics of the selected articles

The demographic detail of the finalized relevant articles is shown in Table 2. Approximately two-thirds of the studies are journal articles (*n* = 19, ≈ 68%) (Wu et al., 2021; Aladhadh et al., 2022; Alahmadi and Alghamdi, 2022; Ayas, 2022; Cao et al., 2022; Dong and Wang, 2022; Du et al., 2022; Feng et al., 2022; He et al., 2022; Malik et al., 2022; Nakai et al., 2022; Nofallah, 2022; Wu H. et al., 2022; Wu Y. et al., 2022; Xin et al., 2022; Zhang N. et al., 2022), whereas 09 studies were conference proceedings (~ 32%) (Wang et al., 2021, 2022; Xie et al., 2021; Zhou and Luo, 2021; de Lima and Krohling, 2022; Liu et al., 2022; Nakai and Han, 2022; Sarker et al., 2022; Zhang N. et al., 2022; Zhao, 2022). Most studies were published in 2022 (*n* = 24, ≈ 86%). Table 2 shows a visualization of the included articles based on the type of studies and year-wise distribution of these studies. The included studies were published in 10 countries; however, most of these studies were from China (*n* = 15, ≈ 54%). The highest number of publications in the last year (2022) shows the growing interest of the research community in using vision transformers for skin cancer and lesion detection.

**Table 2:** Demographic details of the finalized research articles.

	**Number of studies**	
**Year**	2021	04
	2022	24
**Countries**	Brazil	01
	China	15
	India	01
	Japan	02
	Netherlands	01
	Pakistan	01
	Saudi Arabia	02
	Turkey	01
	UK	01
	USA	03
**Type of publications**	Conference papers	09
	Journal papers	19

### 3.3. Skin lesion detection and feature extraction

In the included articles, the researchers made a significant contribution to skin lesion detection and extraction. Wu et al. (2021) used a histogram-based segmentation method and morphological operations (opening-closing and hole-filling) to extract individual tissue slices. In Nofallah (2022), a pre-trained MobileNetv2 was used on dermoscopy images to visualize and extract lesion patches. It generated a 1,280-dimensional patch-wise feature map after global average pooling. The studies (Nakai et al., 2022; Wu H. et al., 2022) integrated an attention layer in the CNN-encoder and the transformer-encoder model for visualizing lesion segmentation. These enhanced deep bottleneck transformer models incorporate self-attention to reproduce the global correlation of features accumulated from standard deep models, which improves skin lesion interpretations. Aladhadh et al. (2022) suggested the Grad-CAM conception technique to produce a heat map of the infected region. In Zhou and Luo (2021), the authors presented a novel mutual attention transformer neural network to extract astute values from multi-modal data for skin lesion diagnosis. They designed a transformer unit composed of self-attention and guided-attention blocks to extract enriched features concurrently. In the researh article (Wu H. et al., 2022), a memory-efficient decoder and feature adaptation module were utilized to improve the feature fusion process between adjacent-level features. This fusion process was achieved by suppressing the non-relevant backgroud noise and energizing the effective channels. This approach helped to enhance the overall performance of the network while minimizing memory usage. In He et al. (2022), individual tissue slices were extracted using a histogram-based segmentation method and other morphological operations (opening-closing and hole filling) and contour-related operations available in OpenCV.

The research article (Wang et al., 2021) reported a boundary aware transformer for lesion visualization, while several studies (Xie et al., 2021; Zhou and Luo, 2021; Ayas, 2022; Du et al., 2022) reported vision transformer-based models for skin lesion visualization. The articles (Alahmadi and Alghamdi, 2022; Ayas, 2022; de Lima and Krohling, 2022; Wang et al., 2022) reported a semi-supervised deep learning model and CNN architecture for retrieving semantic-based information from skin dermoscopy images. The study (Liu et al., 2022) presented a new segmentation-based framework called Intensive Atrous Spatial Transformer Network (IASTrans-Net) based on the intensive atrous spatial pyramid pooling module and atrous convolution for optimum feature accumulation and segmentation. To achieve high-quality segmentation results with good contrast, the study (Malik et al., 2022) presented a hybrid meta-heuristic preprocessor that optimizes the decisive attributes selected for the contrast-improvement transformation function. The researchers in Wang et al. (2022) employed a data fusion approach that involved combining two-stream cascaded feature aggregation modules to effectively assimilate multilevel attributes from two limbs. They also introduced a multi-scale expansion-aware module that leverages feature perception and expansion convolution. This module enables the extraction of high-level features with a broader range of context information, thereby improving the network's perception ability. The research studies (Aladhadh et al., 2022; Wu H. et al., 2022; Xin et al., 2022; Zhao, 2022) reported the use of transformer-based models for skin lesion visualization and underlined information collection. These articles presented a pipeline model that includes a new multimodal transformer. This transformer includes two encoders, one for images and another for metadata, as well as one decoder to merge the data from both sources. To extract complex image features, a vision transformer serves as the backbone of the model. The metadata is considered as labels and is embedded using a newly designed soft label encoder. Additionally, a mutual-attention block is introduced in the decoder section to effectively merge image features and metadata features.

### 3.4. Transformer's role in skin cancer

After analyzing the included studies, it was concluded that vision transformer-based models are significantly proposed for skin cancer detection and lesion segmentation. These transformer-based models are based on architectures that combine CNN-based architectures like ResNet, DenseNet, VGG16, hybrid meta-heuristic preprocessor with different transformer-based designs like multi-scale context transformer (MCT), IASTrans-Net, Swin transformer, Swin Pyramid Aggregation network (SwinPA-Net), and many others. The spatial pyramid transformer (SPT) is reported in four different studies (Alquran et al., 2017; Dong and Wang, 2022; Zhang N. et al., 2022). Similarly, the hybrid models (consist of CNN and transformers) are reported in nine different studies (Cao et al., 2022; Du et al., 2022; Feng et al., 2022; Liu et al., 2022; Malik et al., 2022; Nakai and Han, 2022; Wu H. et al., 2022; Zhang N. et al., 2022; 48). While in the remaining studies, either pipelined models using different transformer architectures or decoder encoder models are used for the segmentation of skin lesions and diagnosis of skin cancer.

Twelve studies (Xie et al., 2021; Zhou and Luo, 2021; Aladhadh et al., 2022; Ayas, 2022; de Lima and Krohling, 2022; Dong and Wang, 2022; Du et al., 2022; Feng et al., 2022; Wu H. et al., 2022; Xin et al., 2022; Zhao, 2022) reported Swin transformer. Three studies (Cao et al., 2022; He et al., 2022; Liu et al., 2022) reported fully transformer network (FTN) in association with SPT for feature extraction using dermoscopy images. The studies (Alahmadi and Alghamdi, 2022; Sarker et al., 2022) reported bidirectional pipelined architecture using CNN and transformer, while the research articles (Nakai and Han, 2022; Nakai et al., 2022) used bottleneck transformer model in association with ResNet50 and DenseNet201 for skin lesion classification.

Only eight studies provided links for publicly available implementation code (Wang et al., 2021, 2022; Alahmadi and Alghamdi, 2022; Ayas, 2022; Cao et al., 2022; de Lima and Krohling, 2022; Dong and Wang, 2022; Malik et al., 2022). Table 3 represents the publicly available code repository for implementing transformer-based models in the included studies. For the development of the transformer-based model, most of the researchers (*n* = 22, ≈ 78%) used PyTorch while six studies (*n* = 6, ≈ 22%) used TensorFlow library as a development and programming tool along with different hardware resources for simulation and experimental purposes. Ten studies (Wang et al., 2021; Alahmadi and Alghamdi, 2022; Cao et al., 2022; He et al., 2022; Liu et al., 2022; Malik et al., 2022; Sarker et al., 2022) reported using NVIDIA GeForce RTX 3090 GPU (24GB memory) and RTX TITAN GPU for training and experimental purposes. Three studies (Wang et al., 2022; Zhang N. et al., 2022; Zhang Y. et al., 2022) used a single NVIDIA-A100 GPU with 10 GB memory and 4 GB of VRAM. Two studies (Ayas, 2022; Cao et al., 2022) trained their models on a single NVIDIA GeForce 2080 GPU with 10 GB memory. Six studies (Aladhadh et al., 2022; de Lima and Krohling, 2022; Wu H. et al., 2022; Xin et al., 2022; Zhao, 2022) used TensorFlow with Keras library on a Core i5-7200u CPU (2.7 GHz) with a main memory of 8 GB and a GeForce GTX 2060 GPU with 6 GB memory for the training and testing of their models. Only one study (Wu H. et al., 2022) used the stochastic gradient descent optimizer, while the rest of the 27 studies used the Adam optimizer (AdamW). Almost all the research articles have reported a learning rate of 0.001 and the number of epochs equal to 200.

**Table 3:** Different transformer-based models with access link.

**S. No**	**Transformer model**	**Code access link**	**References**
1	Scale-aware transformer	https://github.com/meredith-wenjunwu/ScATNet	Cao et al., 2022
2	Transformer model based on wavelet scattering network (ScatNet)	http://group.bmj.com/group/rights-licensing/permissions	Alahmadi and Alghamdi, 2022
3	Fully adaptive transformer network using encoder-decoder architecture (FAT-Net)	https://github.com/SZUcsh/FAT-Net	Ayas, 2022
4	Bottleneck transformed model	http://mlp.sci.yamaguchi-u.ac.jp/index_EN.html	Malik et al., 2022
5	Fully transformer network	https://github.com/Novestars/Fully-Transformer-Network	Dong and Wang, 2022
6	SLT-Net	https://www.github.com/FengKaili-fkl/SLT-Net.git	Wang et al., 2021
7	TransFuse	https://github.com/MIC-DKFZ/nnUNet	Liu et al., 2022
8	Boundary-aware transformer	https://github.com/jcwang123/BA-Transformer	Wang et al., 2022

### 3.5. Evaluation metrics

Multiple performance and validation metrics are reported in the included studies to evaluate the performance of the vision transformer-based models. The most commonly used metrics were recall/sensitivity (reported in n=18 studies), accuracy (*n* = 17 studies), specificity (*n* = 14 studies), dice similarity score (*n* = 12 studies), area under the receiver operating characteristic curve (AUC) values (*n* = 08 studies), and Jaccard similarity index (*n* = 06 studies). Table 4 shows the different performance evaluation metrics used in the included studies.

**Table 4:** Performance evaluation metrics used in the included studies.

**S. No**	**Performance metric**	**Number of studies**	**References**
1	Accuracy	17	Wu et al., 2021; Zhou and Luo, 2021; Aladhadh et al., 2022; Alahmadi and Alghamdi, 2022; Ayas, 2022; Cao et al., 2022; Dong and Wang, 2022; He et al., 2022; Liu et al., 2022; Nakai and Han, 2022; Nakai et al., 2022; Sarker et al., 2022; Wu H. et al., 2022; Wu Y. et al., 2022; Zhang N. et al., 2022; Zhao, 2022
2	Sensitivity/recall	18	Wu et al., 2021; Aladhadh et al., 2022; Alahmadi and Alghamdi, 2022; Ayas, 2022; Cao et al., 2022; Dong and Wang, 2022; Du et al., 2022; Feng et al., 2022; He et al., 2022; Nakai and Han, 2022; Nakai et al., 2022; Nofallah, 2022; Sarker et al., 2022; Wu H. et al., 2022; Wu Y. et al., 2022; Xin et al., 2022; Zhang N. et al., 2022
3	Specificity	14	Wu et al., 2021; Aladhadh et al., 2022; Alahmadi and Alghamdi, 2022; Ayas, 2022; Cao et al., 2022; Dong and Wang, 2022; Feng et al., 2022; He et al., 2022; Nakai and Han, 2022; Nakai et al., 2022; Nofallah, 2022; Wu H. et al., 2022; Wu Y. et al., 2022; Zhang N. et al., 2022
4	F-score	08	Wu et al., 2021; He et al., 2022; Nakai and Han, 2022; Nofallah, 2022; Sarker et al., 2022; Wu H. et al., 2022; Xin et al., 2022
5	Return on investment (ROI)	01	Wu et al., 2021
6	Receiver operating characteristic curve (ROC)	01	Wu et al., 2021
7	Intersection over union (IoU)	05	Wang et al., 2021, 2022; Feng et al., 2022; Wu H. et al., 2022; Zhang N. et al., 2022
8	Dice similarity coefficient	12	Wang et al., 2021, 2022; Alahmadi and Alghamdi, 2022; Cao et al., 2022; Dong and Wang, 2022; Feng et al., 2022; He et al., 2022; Liu et al., 2022; Malik et al., 2022; Wu H. et al., 2022; Zhang N. et al., 2022; Zhang Y. et al., 2022
9	Training and validation loss	02	Aladhadh et al., 2022; Zhao, 2022
10	AUC values	08	Wu et al., 2021; Xie et al., 2021; Zhou and Luo, 2021; de Lima and Krohling, 2022; He et al., 2022; Wu H. et al., 2022; Xin et al., 2022
11	Label ranking average precision (LRAP)	01	Zhou and Luo, 2021
12	Jaccard similarity index (JI)	06	Cao et al., 2022; Dong and Wang, 2022; He et al., 2022; Liu et al., 2022; Malik et al., 2022; Zhang N. et al., 2022
13	Balanced accuracy	02	Ayas, 2022; de Lima and Krohling, 2022
14	TF values	03	Cao et al., 2022; Wang et al., 2022; Wu H. et al., 2022
15	Confusion matrix	02	Wu H. et al., 2022; Zhao, 2022
16	Mean Dice coefficient (mDice)	02	Du et al., 2022; Zhang N. et al., 2022
17	Mean absolute error (MAE)	01	Du et al., 2022
18	Mean intersection over union (mIoU)	02	Du et al., 2022; Zhang N. et al., 2022
19	Relative volume difference (RVD)	01	Feng et al., 2022
20	Precision	06	Xie et al., 2021; Aladhadh et al., 2022; Dong and Wang, 2022; Sarker et al., 2022; Xin et al., 2022
21	Pixel-wise accuracy	01	Zhang N. et al., 2022

Four studies (Zhou and Luo, 2021; Aladhadh et al., 2022; Nakai and Han, 2022; Nakai et al., 2022) reported the use of more than 10,000 (<12,000) dermoscopy images, and nine studies (Xie et al., 2021; Alahmadi and Alghamdi, 2022; Dong and Wang, 2022; Du et al., 2022; Liu et al., 2022; Malik et al., 2022; Wang et al., 2022; Wu H. et al., 2022; Zhang N. et al., 2022) reported the use of more than 2,000 (<3,000) dermoscopy images for training and testing the diagnosing model. In two studies, the number of images used for training and validation purposes was between 200 and 500. In the included studies, 11 articles reported data splitting into training and test sets, while the same number of studies (11 studies) reported splitting the data into training, validation, and test sets. Other studies reported the use of a k-fold cross-validation mechanism for evaluation purposes; for example, 5-fold cross-validation was reported in two studies (Table 5). External evaluation by human experts was reported in only two studies (Wu et al., 2021; Nofallah, 2022). The study in Malik et al. (2022) selected images for training and test sets from four different datasets (ISIC-2016, ISIC-2017, ISIC-2018, and PH^2^) for experimental work in the form of three different groups. Group 1 contained 200 images from PH^2^ and 900 images from ISIC-2016 for the training set, while the test set contained 379 images from the ISIC-2016 testing dataset. For group 2, all the images of the ISIC-2017 dataset were selected by choosing 2000 training and 600 testing images. For group 3, the ISIC-2018 images were divided by selecting 2076 images for training and 518 images for the test set.

**Table 5:** Evaluation mechanisms proposed in the finalized relevant articles.

**S. No**	**Evaluation strategy**	**Number of articles**	**References**
1	Training and test split	11	Ayas, 2022; de Lima and Krohling, 2022; Du et al., 2022; Liu et al., 2022; Nakai and Han, 2022; Nakai et al., 2022; Nofallah, 2022; Wang et al., 2022; Wu H. et al., 2022; Wu Y. et al., 2022; Zhang N. et al., 2022
2	Training, validation, and test split	11	Wang et al., 2021, 2022; Wu et al., 2021; Xie et al., 2021; Zhou and Luo, 2021; Aladhadh et al., 2022; Alahmadi and Alghamdi, 2022; Dong and Wang, 2022; Liu et al., 2022; Wu H. et al., 2022; Zhang N. et al., 2022
3	Five-fold cross validation method	01	Xu et al., 2022
4	Four-fold cross validation method	01	Cao et al., 2022
5	Group-based selection (random selection of images for training, validation, and test sets from different datasets).	01	Malik et al., 2022

In the included studies, multiple benchmark models are reported for the comparison and performance evaluation purposes. Different variations of UNet (UNet+, UNet++, ResUNet, AttU-Net, R2U-Net UNet3+, etc.) are used in eight studies (Alquran et al., 2017; Ayas, 2022; Liu et al., 2022; Nakai and Han, 2022; Wang et al., 2022; Wu H. et al., 2022; Zhang N. et al., 2022). Multiple architectures of CNN models (Res50, RAN50, SEnet50, ARL-CNN50, etc.) are used in twelve research articles (Alquran et al., 2017; Zhou and Luo, 2021; Ayas, 2022; de Lima and Krohling, 2022; Du et al., 2022; Feng et al., 2022; Malik et al., 2022; Wang et al., 2022; Wu H. et al., 2022; Zhang N. et al., 2022; Zhang Y. et al., 2022; World Health Organization, 2023). Numerous transformer-based models such as TransFuse, multi-model transformers, FAT-Net, SwinUNet transformers are reported in Wang et al. (2021), Zhou and Luo (2021), Du et al. (2022), Feng et al. (2022), Nakai and Han (2022), Wu H. et al. (2022), Zhang N. et al. (2022), World Health Organization (2023). Only one study (World Health Organization, 2023) has reported ISIC-2017 winner-1and ISIC-2017 winner-2 as benchmark techniques for their skin cancer detection model.

### 3.6. Datasets

**Figure 2:**
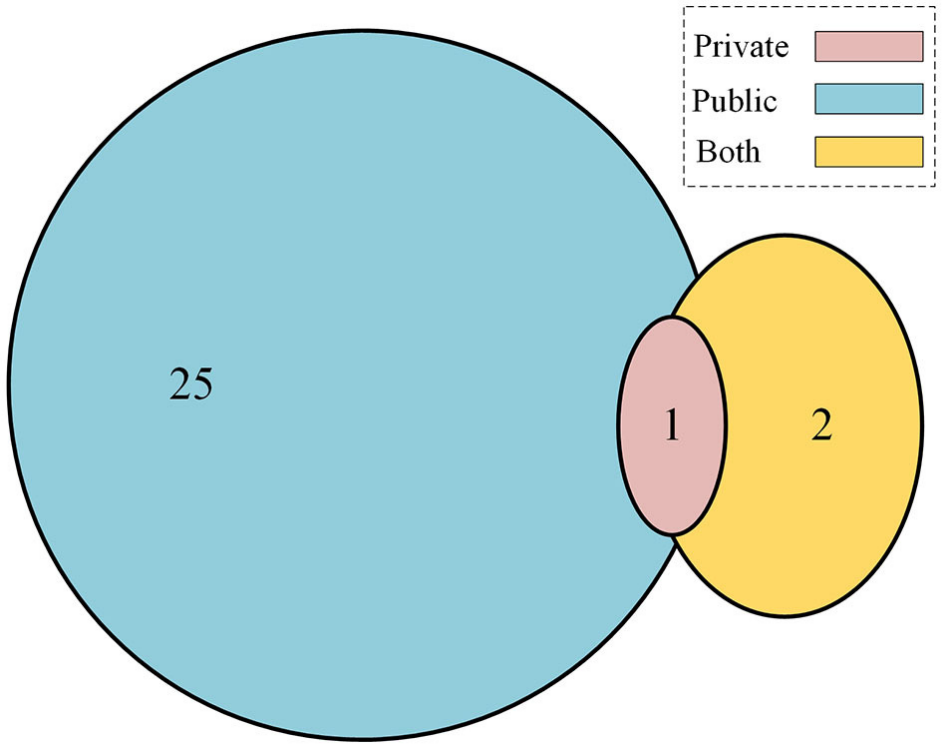
Contribution of public vs. private datasets for skin cancer.

In the final set of included studies, 25 articles (Wang et al., 2021, 2022; Xie et al., 2021; Zhou and Luo, 2021; Aladhadh et al., 2022; Alahmadi and Alghamdi, 2022; Ayas, 2022; Cao et al., 2022; de Lima and Krohling, 2022; Dong and Wang, 2022; Feng et al., 2022; He et al., 2022; Liu et al., 2022; Malik et al., 2022; Nakai and Han, 2022; Nakai et al., 2022; Nofallah, 2022; Sarker et al., 2022; Wu H. et al., 2022; Wu Y. et al., 2022; Xin et al., 2022; Zhang N. et al., 2022; Zhao, 2022) used publicly available datasets for training, validation, and testing of the transformer-based models. All these datasets contain dermoscopy images that were preprocessed to remove other unwanted information, and then classification tasks were reported accordingly. One study (Zhang N. et al., 2022) used a private dataset only, and two studies (Wu et al., 2021; Du et al., 2022) reported using both private and public datasets, as shown in the Venn diagram in Figure 2. Table 6 shows the list of publicly available datasets along with access. Furthermore, some studies (Ayas, 2022; de Lima and Krohling, 2022; Nofallah, 2022) followed a random selection mechanism for training the model with dermoscopy images, while some studies followed a percentage distribution of images for training and validation purposes, such as the study in Aladhadh et al. (2022) selected 70% of the data for training, 20% for validation, and 10% for testing the model. The research study (Nakai et al., 2022) used a ratio of 80% data for training and 20% data for testing purposes. The study (Zhou and Luo, 2021) used 80% data for training, 10% for validation, and 10% for testing the classification model. The articles (Wang et al., 2022; Wu H. et al., 2022) distributed the ISIC-2018 dermoscopy images dataset into training, validation, and test sets with 80%, 10%, and 10%, respectively. The study (Zhang N. et al., 2022) selected 70% images for training, 10% for validation, and 20% for testing the model, while the study (Xu et al., 2022) divided the entire dataset randomly divided into five folds on the patient level with a distribution ratio of 70% data for the training set, 10% for the validation set, and 20% for the testing set.

**Table 6:** Datasets for skin cancer.

**S. No**	**Name of dataset**	**Access link**	**Reference**
1	Kvasir and CVC-ClinicDB	https://github.com/MIC-DKFZ/nnUNet	Zhang N. et al., 2022
2	ISBI-2017	https://www.kaggle.com/datasets/soumikrakshit/isbi-challenge-dataset	Dong and Wang, 2022
3	PAD-UFES-20	https://github.com/labcin-ufes/PAD-UFES-20	de Lima and Krohling, 2022
4	ISIC-2019	https://www.kaggle.com/datasets/andrewmvd/isic-2019	Ayas, 2022
5	Derm7pt dataset	https://github.com/jeremykawahara/derm7pt	Nofallah, 2022
6	M-Path	https://github.com/meredith-wenjunwu/ScATNet	Wu et al., 2021; Nofallah, 2022
7	ISIC-2016	https://www.kaggle.com/datasets/soumikrakshit/isbi-challenge-dataset	Wang et al., 2021; Cao et al., 2022; Dong and Wang, 2022; Feng et al., 2022; Malik et al., 2022; Wu H. et al., 2022
8	PH^2^	https://www.kaggle.com/datasets/synked/ph2-modified	Wang et al., 2021; Alahmadi and Alghamdi, 2022; Cao et al., 2022; Malik et al., 2022; Wu H. et al., 2022; Zhang N. et al., 2022
9	HAM10000	https://www.kaggle.com/datasets/kmader/skin-cancer-mnist-ham10000	Aladhadh et al., 2022; Nakai and Han, 2022; Nakai et al., 2022; Sarker et al., 2022; Xin et al., 2022; Zhao, 2022
10	ISIC-2018	https://challenge.isic-archive.com/landing/2018/	Wang et al., 2021; Alahmadi and Alghamdi, 2022; Cao et al., 2022; Dong and Wang, 2022; Du et al., 2022; Feng et al., 2022; He et al., 2022; Liu et al., 2022; Malik et al., 2022; Wu H. et al., 2022; Wu Y. et al., 2022
11	ISIC-2017	https://www.kaggle.com/datasets/awsaf49/isic-2017	Xie et al., 2021; Zhou and Luo, 2021; Alahmadi and Alghamdi, 2022; Dong and Wang, 2022; Feng et al., 2022; Liu et al., 2022; Malik et al., 2022; Wu H. et al., 2022; Zhang N. et al., 2022
12	LIVis Dataset	The LIVis dataset comprises clinical data that is private and was collected during six TME surgeries performed on six patients using surgical robots.	Du et al., 2022

In some studies, researchers used a hybrid approach combining different datasets for training and testing. For example, Cao et al. (2022) followed the K-fold mechanism for selecting 900 images from the ISIC2016 dataset for training, and 200 images from the PH^2^ dataset for testing. Similarly, Malik et al. (2022) selected four different datasets for experimental work and divided these datasets into varying training and test sets in the form of three different groups. (1) In the first group, they selected a combination of 200 images from PH^2^ and 900 images from ISIC-2016 for training. While for testing, they selected 379 images from the ISIC-2016 testing dataset. (2) For group 2, all the images of the ISIC-2017 dataset are selected by choosing 2000 training and 600 testing images. (3) For group 3, the selected ISIC-2018 contains a total of 2594 dermoscopy images due to the missing masks of test images; they further divided the dataset into 2076 training images and 518 testing images. In the articles Xie et al. (2021), Alahmadi and Alghamdi (2022), Cao et al. (2022), Dong and Wang (2022), Liu et al. (2022), Nakai and Han (2022), Wang et al. (2022), Wu H. et al. (2022), random training, validation, and test sets are selected from different databases such as from ISIC-2017, the authors used 2,000 images for the training set, 150 images for the validation set, and 600 images for the test set. While from ISIC-2018, they selected 2,594 images for the training set, 100 images for the validation set, and 1,000 images for the test set. As the available testing datasets were unlabeled, the research article (Alahmadi and Alghamdi, 2022) randomly selected 1,815 images for the training set, 259 for the validation set, and 520 for the test set.

In the studies included, the commonly used dataset is the ISIC 2018 dataset, which was reported in nine studies (Table 6). Across different studies, various versions of the ISIC datasets (ISIC-2016, ISIC-2017, ISIC-2018, and ISIC-2019) were used in 24 articles. The Human Against Machine (HAM10000) dataset was reported in two studies. The dataset consists of 240 skin biopsy images featuring hematoxylin and eosin (H&E) staining. It was obtained as a segment of the MPATH study (R01CA151306) and was authorized by the Institutional Review Board at the University of Washington under protocol number STUDY00008506.

## 4. Discussions

### 4.1. Principle results

During the data synthesis process, we found that most studies were published in 2021 and 2022. This trend is not surprising, as the use of vision transformers for skin cancer applications has only recently gained popularity. Over half of the studies were published in China (15 studies ≈ 54%). The second closest number of studies from one country was three (11%) published in the USA. In comparison, two studies were published in Saudi Arabia and Japan each, while the remaining countries published only one study each.

In almost half of the studies, the Swin transformer and other variants of vision transformers are used for lesion segmentation and skin melanoma diagnosis, often in conjunction with GANs for data synthesis and augmentation. Many studies also utilized CNN architectures, such as ResNet, DenseNet, and VGG16, for semantic-based information retrieval from dermoscopy images. Transformers have also been used to enhance image quality, including super-resolution (reported in 12 studies) and noise removal (reported in 21 studies). While transformers are widely used for disease diagnosis, their use is typically focused on specific lesion boundary detection or semantic-based information retrieval.

The term “semantic information” in this review is used in a broad sense and encompasses various feature extraction techniques reported in the studies included for the extraction of both global and local features (detailed information) from dermoscopy images of skin lesions. These semantic-based features (feature maps) were then utilized to improve diagnosis, such as detecting skin cancers or segmenting skin lesions in dermoscopy images for accurate treatment.

The most popular architecture choice among the studies included was the hybrid or pipelined design that used transformer-transformer or transformer-CNN architectures (about 14 studies). Another popular choice was a fully transformer network, which learns long-range contextual information for skin lesion analysis through hierarchical transformer calculating attributes using SPT. Many studies only made minor changes to the architecture or did not provide sufficient information to the modification transplanted, so it is beyond the range of this scoping review to evaluate all the transformer models. The focus of this review is to analyze the capabilities of different transformer-based models for melanoma detection based on the content presented in the relevant articles.

The most common methods for cross-validation in the studies included are; (1) training and testing and (2) training, validation, and testing. However, real-time testing and validation of the model's performance is still awaited and should be urged in future recommendations.

### 4.2. Challenges

After synthesizing the finalized relevant articles, some of the primary challenges observed on the applications for vision transformers for skin cancer are listed below:

- Intrinsic visual ambiguities—intrinsic visual ambiguities displayed in multi-modal imaging data for skin tumors pose significant challenges in achieving precise diagnoses, particularly at early diagnosis using vision transformers.
- Irregular skin lesion shape—researchers face a significant challenge in developing an ideal segmentation model for skin cancer due to the full-scale interpretations and non-uniforms shapes of skin lesions. The indistinct confines between skin lesions and adjacent tissue can further compound the difficulty in achieving accurate segmentation. Traditional non-adaptive models have constraints in capturing global contextual information and tend to deliver subpar segmentation outcomes.
- Use of traditional CNN models—many studies have utilized CNN architectures to extract meaningful information from dermoscopy images for skin lesion segmentation. However, the use of convolution layers to capture local information may not be sufficient for precise segmentation in complex, low-contrast datasets, as this approach ignores pixel relationships. Moreover, the locality of the convolution operator inherent in CNNs can limit their ability to capture long-range dependencies and contextual information.
- Gradient local features—transformers excel in modeling global features, but their capacity to extract fine-grained local features is limited.
- Smaller images dataset problem—transformers have shown poor performance with smaller image datasets (de Lima and Krohling, 2022). Conversely, CNN architectures perform well with comparatively smaller datasets.
- Explainable AI (XAI)—XAI refers to the ability of artificial intelligence systems to provide understandable and transparent explanations for their decisions and actions. In the medical field, XAI enhances transparency, accountability, trust, and the ability to identify and correct errors, thus, increasing acceptance of the AI-based methods. However, in the included studies, we did not find reporting of explainability aspect of vision transformer-based approaches for skin cancer application.

### 4.3. Research and practical implications

Most of the studies included in the review reported results on openly accessible datasets. The incredibly used datasets among the research community are the ISIC dataset (ISIC-2016, ISIC-2017, ISIC-2018, ISIC-2019) and the HAM10000 dataset. To facilitate the reproducibility of the existing models and methods, it would be beneficial for the researchers to dispense the corresponding programming codes/software for the results published in the contained studies. However, some studies did not provide the code, which limits the chance for real-life-scenarios validation of the claims crafted in the contained studies.

No smart framework was found to be enacted on mobile devices in the studies selected in this review. The computational requirements of vision transformers and the memory resources needed for dermoscopy imaging data could be the justifications for the restricted transfer of the developed applications to the mobile phones. Future research will likely make it possible to implement these methods on mobile devices, connecting them to servers to perform diagnoses at the patient's doorstep. This will not only help to reduce the burden on healthcare centers but also assist practitioners in providing treatment to patients at home and recommending medications accordingly.

Numerous studies examined in this review employed publicly available data on skin cancer, mainly originating from evolved economies. Regrettably, there is a paucity of medical imaging data from progressing economies. Consequently, developing smart applications for cancer diagnosing using such data for training and validation process may not be suitable for populations with differing economic and demographic backgrounds, owing to inadequate representation in the data. To enhance AI techniques for clinical applications such as diagnosis, prognosis, and lesion segmentation in dermoscopy images, it is imperative to incorporate dermoscopy imaging data from a diverse range of locations.

## 5. Strengths and limitations

The key strengths and limitations of this scoping review are described in the following subsections.

### 5.1. Strengths

Some review articles have been published on the applications of deep learning and other machine learning models in skin cancer segmentation and detection. However, these review articles are limited to CNN-based models or shallow architectures and lack recent studies, as shown in Table 1. To the best of our knowledge, this is the first review of the applications of vision transformers in skin cancer applications. This review included all studies that used vision transformers for skin cancer, making it the most comprehensive review on the topic. It will assist the readers to understand the potential of transformers for the segmentation of skin lesions and for improving the diagnosis of skin cancer.

For this scoping review, we adhered to the PRISMA-ScR scientific review guidelines (Pathan et al., 2018). Our search for published studies spanned several key databases in the fields of health sciences, engineering, and technology to ensure that we captured as many relevant studies as possible. To prevent any bias in our study selection process, we employed a strategy that involved two independent reviewers conducting the study selection and data extraction, and a third reviewer validating the screening and data extraction. Moreover, we compiled an exhaustive inventory of publicly accessible datasets related to skin cancers, which would be helpful for readers in identifying high-quality datasets for skin cancer imaging. As a result, this review serves as a valuable resource for the skin cancer research community.

### 5.2. Limitations

While every effort has been made to ensure the validity of this review, some limitations might be associated with it. The literature search was conducted on only four databases, which may have resulted in the exclusion of studies not available in these databases. Additionally, only studies published in English were included, potentially omitting relevant studies published in other languages. The studies were categorized into major applications, but there may be partial overlap between categories and the categorization may not fully reflect the nature of the applications. Furthermore, this review did not evaluate the claims made regarding the diagnosis of skin cancer or the quality of synthesized dermoscopy data, as this was beyond the scope of the review.

## 6. Conclusion and recommendations

In this scoping review, we analyzed 28 studies that utilized transformers for skin cancer diagnosis using dermoscopy images. The ISIC and HAM10000 datasets were the most popular openly accessible datasets used in these studies. Additionally, we noted that the hybrid or pipelined designs that use transformer-transformer or transformer-CNN architectures were the most commonly used architectures. Furthermore, most of the studies published results on openly accessible datasets and validated their models using training and testing or training, validation, and testing methods. However, we found a lack of implementation of these models on mobile devices and a need for more data from diverse locations. It is important to emphasize the need for the reproducibility of results by making the software and codes for these studies available. Collaboration between computer scientists and clinicians is also crucial for the progress on skin cancer diagnosis. Furthermore, standardizing the comparison protocols for the different transformer architectures used for melanoma detection using dermoscopy images will be beneficial for the advancement of this field.

## Data availability statement

The original contributions presented in the study are included in the article/Supplementary material, further inquiries can be directed to the corresponding author.

## Author contributions

SK and HA contributed to conceptualization and reviewed and edited the draft. The data extraction process was performed SK and HA and verified by ZS. ZS supervised the study. SK contributed to writing original draft. All authors read and approved the final manuscript.

## Supplementary material

Supplementary Table 1 PRISMA-ScR checklist.

Supplementary Table 2 Search strategy.

Supplementary Table 3 Data extraction form.

Supplementary Files Characteristics and materials in the studies.
