# Supplementary material for: Identifying the role of vision transformer for skin cancer—A scoping review
Source: Front Artif Intell. 2023 Jul 17;6:1202990. doi: 10.3389/frai.2023.1202990 (PMC10388102; doi:10.3389/frai.2023.1202990)
Supplement: Supplementary Table 1 — PRISMA-ScR checklist. [file Table_1.DOCX]

**ELECTRONIC SUPPLEMENTARY MATERIAL**

**Identifying the Influence of Vision Transformers and Deep Learning Models for Skin Cancer Detection—A Scoping Review**

**Supplementary Table 1: PRISMA-ScR Checklist**

| SECTION | ITEM | PRISMA-ScR CHECKLIST ITEM | Reported in Section |
| --- | --- | --- | --- |
| TITLE | | | |
| Title | 1 | Identify the report as a scoping review. | Title page |
| ABSTRACT | | | |
| Structured summary | 2 | Provide a structured summary that includes (as applicable): background, objectives, methods, results, and conclusions that relate to the review questions and objectives. | Abstract |
| INTRODUCTION | | | |
| Rationale | 3 | Describe the rationale for the review in the context of what is already known. Explain why the review questions/objectives lend themselves to a scoping review approach. | Introduction |
| Objectives | 4 | Provide an explicit statement of the questions and objectives being addressed with reference to their key elements (e.g., population or participants, concepts, and context) or other relevant key elements used to conceptualize the review questions and/or objectives. | Introduction |
| METHODS | | | |
| Protocol and registration | 5 | Indicate whether a review protocol exists; state if and where it can be accessed (e.g., a Web address); and if available, provide registration information, including the registration number. | Methods |
| Eligibility criteria | 6 | Specify characteristics of the sources of evidence used as eligibility criteria (e.g., years considered, language, and publication status), and provide a rationale. | Methods |
| Information sources | 7 | Describe all information sources in the search (e.g., databases with dates of coverage and contact with authors to identify additional sources), as well as the date the most recent search was executed. | Methods |
| Search | 8 | Present the full electronic search strategy for at least 1 database, including any limits used, such that it could be repeated. | Methods and S2 Table |
| Selection of sources of evidence | 9 | State the process for selecting sources of evidence (i.e., screening and eligibility) included in the scoping review. | Methods |
| Data charting process | 10 | Describe the methods of charting data from the included sources of evidence (e.g., calibrated forms or forms that have been tested by the team before their use, and whether data charting was done independently or in duplicate) and any processes for obtaining and confirming data from investigators. | Methods and S4 Table |
| Data items | 11 | List and define all variables for which data were sought and any assumptions and simplifications made. | Methods |
| Critical appraisal of individual sources of evidence | 12 | If done, provide a rationale for conducting a critical appraisal of included sources of evidence; describe the methods used and how this information was used in any data synthesis (if appropriate). | N/A |
| Synthesis of results | 13 | Describe the methods of handling and summarizing the data that were charted. | Methods |
| RESULTS | | | |
| Selection of sources of evidence | 14 | Give numbers of sources of evidence screened, assessed for eligibility, and included in the review, with reasons for exclusions at each stage, ideally using a flow diagram. | Results |
| Characteristics of sources of evidence | 15 | For each source of evidence, present characteristics for which data were charted and provide the citations. | Results |
| Critical appraisal within sources of evidence | 16 | If done, present data on critical appraisal of included sources of evidence (see item 12). | N/A |
| Results of individual sources of evidence | 17 | For each included source of evidence, present the relevant data that were charted that relate to the review questions and objectives. | Results and S5 Table |
| Synthesis of results | 18 | Summarize and/or present the charting results as they relate to the review questions and objectives. | Results |
| DISCUSSION | | | |
| Summary of evidence | 19 | Summarize the main results (including an overview of concepts, themes, and types of evidence available), link to the review questions and objectives, and consider the relevance to key groups. | Discussion |
| Limitations | 20 | Discuss the limitations of the scoping review process. | Discussion |
| Conclusions | 21 | Provide a general interpretation of the results with respect to the review questions and objectives, as well as potential implications and/or next steps. | Conclusion |
| FUNDING | | | |
| Funding | 22 | Describe sources of funding for the included sources of evidence, as well as sources of funding for the scoping review. Describe the role of the funders of the scoping review. | N/A |

**Supplementary Table 2: Search Strategy**

1. **Search sources:**

- Electronic databases: Pubmed, Scopus, IEEE Xplore, Google Scholar. (Medline covered in Pubmed)
- Reference list checking for additional relevant papers.

1. **Search terms:**

Intervention-related terms: Vision Transformers OR Transformers

Application-related terms: Cancer OR cancer imaging OR melanoma OR lesion OR skin OR carcinoma OR melanocyte cancer.

Years: 2017 to 2022

Search dates: 20 November 2022 to 07 January 2023

Initial raw search results: 298 studies

| **Database** | **Search strategy** | **Hits** |
| --- | --- | --- |
| IEEExplore | ((vision transformer) OR (transformer)) AND ((skin cancer) OR (skin) OR (lesion) OR (melanoma) OR (carcinoma) OR (Melanocyte cancer)) | 47 |
| Pubmed | ((vision transformer) OR (transformer)) AND ((skin cancer) OR (skin) OR (lesion) OR (melanoma) OR (carcinoma) OR (Melanocyte cancer)) | 94 |
| Scopus | TITLE-ABS-KEY ( ( ( vision  AND  transformers )  OR  ( transformers ) )  AND  ( ( skin  AND  cancer )  OR  ( skin )  OR  ( lesion )  OR  ( melanoma )  OR  ( carcinoma )  OR  ( melanocyte  AND  cancer ) ) )  AND  ( LIMIT-TO ( PUBYEAR ,  2023 )  OR  LIMIT-TO ( PUBYEAR ,  2022 )  OR  LIMIT-TO ( PUBYEAR ,  2021 )  OR  LIMIT-TO ( PUBYEAR ,  2020 )  OR  LIMIT-TO ( PUBYEAR ,  2019 )  OR  LIMIT-TO ( PUBYEAR ,  2018 )  OR  LIMIT-TO ( PUBYEAR ,  2017 ) )  AND  ( EXCLUDE ( DOCTYPE ,  "re" )  OR  EXCLUDE ( DOCTYPE ,  "le" ) )  AND  ( LIMIT-TO ( LANGUAGE ,  "English" ) ) | 57 |
| Google Scholar | ((vision transformer) OR (transformer)) AND ((skin cancer) OR (skin) OR (lesion) OR (melanoma) OR (carcinoma) OR (Melanocyte cancer)) | 100 |

**Supplementary: Study Eligibility Criteria**

1. Population

*Inclusion*: People who had undergone clinical procedures such as MRI, CT or biopsy for identification of skin cancer. No restrictions regarding their age, gender, and ethnicity.

Exclusion: People who had performed clinical visits but did not go through diagnosis procedures other than check-up physicians.

1. Intervention

*Inclusion*: Vision Transformers for medical images, for purpose like skin cancer diagnosis, prognosis, segmentation, noise adaptation, classification – ultimately contributing to AI for skin cancer applications.

*Exclusion*: Remove studies with other AI methods only, such as Convolutional Neural Networks or Generative Adversarial Network.

*Inclusion*: Modality of data covers medical images of all modalities such as X-Ray or Mammograms, CT, MRI, Histopathology images.

*Exclusion*: Remove studies with non-image data (for example, text data, EHRs, physiological signals).

1. Comparator: no restrictions applied

1. Outcome: Diagnosis/Detection, Prognosis or survival prediction, Segmentation, Classification, Grading.

1. Language of study

*Inclusion*: English

*Exclusion*: any other language

1. Study design: No restrictions applied

1. Year of study: 2017-2022

1. Type of publications:

*Inclusion*: Peer-reviewed articles, theses, dissertations, conference proceedings, book chapters.

*Exclusion*: Reviews, preprints, conference abstracts, proposals, short letters, editorials, commentaries and non-peer reviewed pre-prints.

**Study selection process**

- Screening titles and abstracts, and then reading full texts.
- Include studies via backward and forward referencing.
- This process is conducted by two reviewers (HA & SK)

**Data extraction process**

- Data regarding the study, population, intervention, comparator, the outcome will be extracted using Excel
- This process is conducted by two reviewers (HA & SK)

**Data synthesis**

- Narrative synthesis

**Data Extraction Fields**

Data extraction fields will be provided in the Excel sheet.

**Supplementary Table 3: Data extraction form**

| **Concept** | **Definition** |
| --- | --- |
| **Study Characteristics** |  |
| ID | Unique ID assigned to each study |
| Author | The first author of the study. |
| Year | The year in which the study was published |
| Country of publication | Affiliation of the first author of the study. |
| Publication type | Journal or conference or book chapter |
| Conference name | Name of the conference where the study was published |
| Journal name | Name of the journal where the study was published |
| **Transformer models** |  |
| Tasks addressed in the study | What are the applications addressed in the study (e.g., segmentation, diagnosis, detection)? |
| Purpose of using transformer | What was the specific purpose of using transformer (e.g., for lesion detection and segmentation, skin melanoma detection, etc.)? |
| Hardware and software resources used for development | What were different software and hardware-based resources used for the development of different transformer-based models. |
| Type of Transformers | What was the architecture of transformers that were used (e.g., Swin transformer, Vision Transformer, Fully transformer network, etc.)? |
| Key changes in transformer architectures | Did the authors report fundamental changes to the architecture of the transformer models (applied pipelined models by combining encoder-decoder models, transformer-CNN models, transformer-transformer architectures, etc.)? |
|  |  |
| **Dataset** **Characteristics** |  |
| Type of data sources | Public or private |
| Name of the data source if available | The specific name of the dataset or the source (for example, GitHub, BMJ, or Kaggle) |
| Data source (full URL) | Mention full URL of the dataset |
| Dataset size (Number of subjects or individuals) | For how many individuals the data is recorded? |
| Dataset size (number of images) | What is the total number of images in the dataset? |
| Training set | What is the number of images in the training set? |
| Validation set size | What is the number of images in the validation set? |
| Testing set size | What is the number of images in the test set? |
| **Evaluation** |  |
| Type of validation | What type of validation the authors used? (e.g., train test split, k-fold cross validation, external validation) |
| Evaluation metrics | Metrics of evaluation (for example, Accuracy, Precision, Recall, Dice score, F1 score, Sensitivity, Specificity, etc.) |
| Study aim | One statement summary of the work. |
